# Pristine corn kernels as a pH-responsive biosorbent for selective removal of cationic and anionic dyes

**DOI:** 10.1186/s13065-025-01649-1

**Published:** 2025-10-29

**Authors:** Noha A. Abd-Rabo, Asmaa A. Serage, Elsayed R. H. El-Gharkawy, Magda A. Akl

**Affiliations:** Department of Chemistry, Faculty of Science, Mansoura University, Mansoura, 35516 Egypt

**Keywords:** Corn kernels, Biosorbents, AG20, CV, Adsorption, Wastewater

## Abstract

Wastewater treatment is of utmost importance in providing equitable and safe drinking water. The present study uses pristine corn kernel (CK) biosorbent to remove the anionic Acid Green 20 (AG20) and the cationic Crystal Violet (CV) dyes from different water samples. The CK biosorbent was thoroughly characterized with elemental analysis, Scanning electron microscopy (SEM), N_2_ adsorption/desorption isotherm, Brunauer–Emmett–Teller (BET), Fourier transform infrared spectroscopy (FTIR), point of zero charge (pH_PZC_), and Thermogravimetric analysis (TGA). The adsorption of dyes onto CK biosorbent was studied in batch and column modes. The experimental variables influencing the adsorption process were evaluated including the dose of CK biosorbent, contact time, pH, temperature, and initial dye concentration. The adsorption process is best described by the pseudo-second-order kinetic and Langmuir isotherm models with maximum adsorption capacities of 85.74 and 61.1 mg/g for AG20 and CV, respectively. The thermodynamic studies revealed that the adsorption of AG20 and CV dyes onto CK biosorbent is spontaneous and endothermic. The CK biosorbent can be regenerated successfully up to five cycles. Furthermore, the antibacterial activity studies of CK before and after dye binding were performed to assess the toxicity of these materials. The CK biosorbent was successfully used to remove CV and AG20 from real water samples with a recovery % more than 90%. The plausible adsorption mechanism of AG20 and CV onto CK biosorbent is proposed due to pore diffusion, electrostatic interactions, π–π interactions, n–π interactions and hydrogen bonds.

**Graphical abstract:**

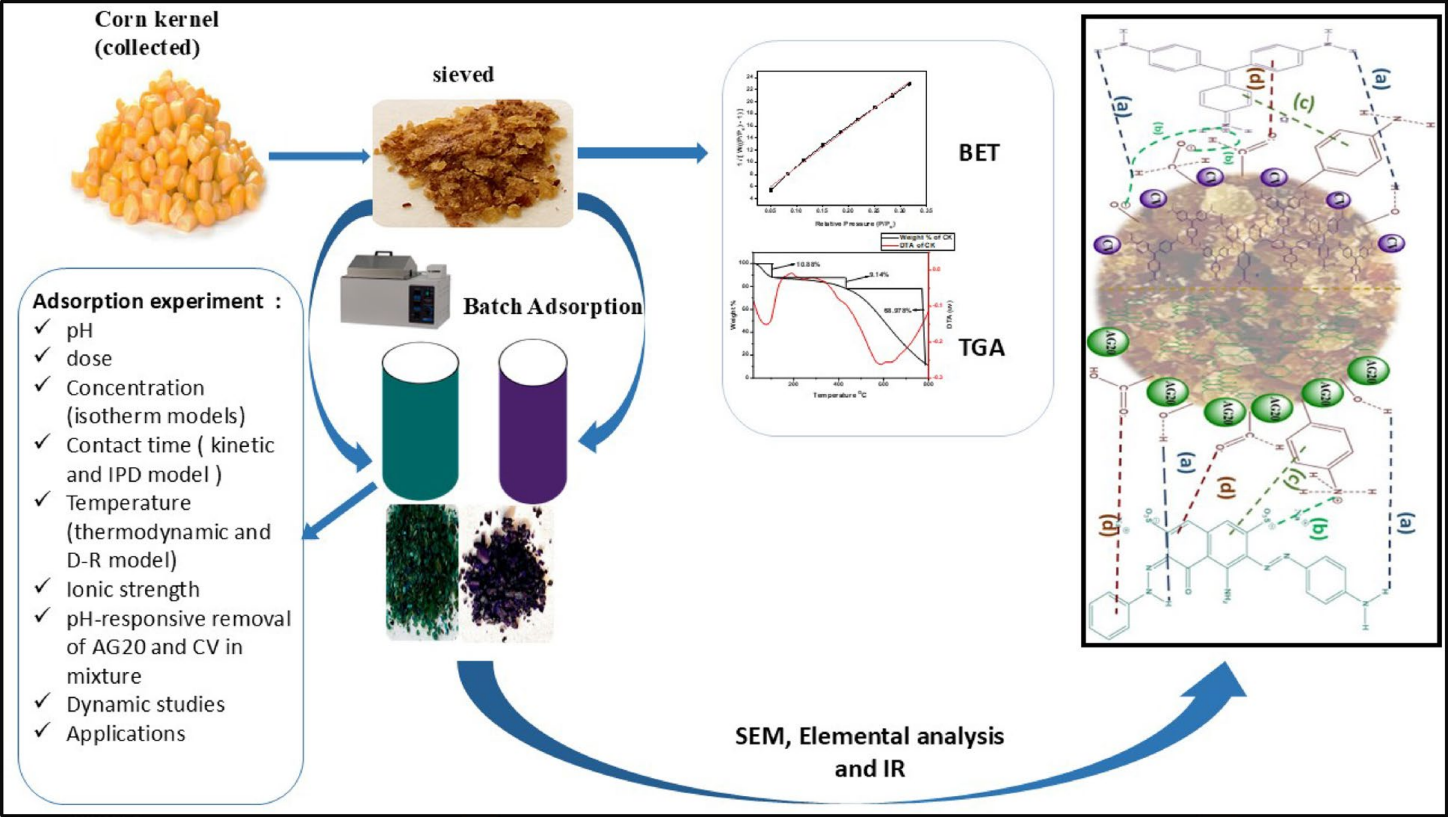

**Supplementary Information:**

The online version contains supplementary material available at 10.1186/s13065-025-01649-1.

## Introduction

Water is essential for all life forms and remains vital for human survival. However, only 2.5% of Earth’s water is freshwater with the remaining 97.5% being saltwater, requiring purification before use [1, 2]. Rapid urbanization has increased industrial wastewater discharge contributing to severe environmental pollution [3]. Among the most persistent pollutants are synthetic dyes and heavy metals, which are resistant to biodegradation due to their complex structures. Water contamination significantly threatens ecosystems and human health [4]. Ensuring access to clean water and a non-toxic environment remains a global priority, particularly in light of increasing freshwater scarcity [5]. Toxic substances such as synthetic dyes in wastewater, harm aquatic life and the environment [6]. Although no specific WHO limits are currently available for the discharge of synthetic dyes such as AG20 and CV, international environmental standards emphasize minimizing the release of persistent organic pollutants due to their toxicity, bioaccumulation, and long-term environmental impacts. These concerns reinforce the urgency of developing effective treatment strategies for dye-containing wastewater.

Dyes are colored compounds commonly used in textiles, leather, paper, and fibers. They are generally classified as cationic (basic) or anionic (acidic, direct, and reactive). Anionic dyes include compounds such as Acid Green 20 (AG20) and Methyl Orange (MO), while Crystal Violet (CV) and Methylene Blue (MB) represent common cationic dyes [7].

AG20 is a non-biodegradable azo dye extensively used in the textile and printing industries due to its high stability against light, sweat, and washing. CV is widely used as a biological stain, especially in Gram staining and light microscopy [8–10]. Prolonged exposure to these synthetic dyes poses significant health risks including carcinogenicity, mutagenicity, and potential damage to the cardiovascular, nervous and renal systems [11]. Rapid industrialization has surged the volume of dye-containing wastewater creating a severe burden on water resources. The shortage of clean water for human consumption has become critical prompting the development of various conventional and advanced wastewater treatment technologies [12].

The conventional methods of water treatment include advanced oxidation processes [13], electrochemical processes [14], membrane separation [15], coagulation-flocculation [16], evaporation [17], flotation [18], photocatalytic degradation/photodegradation [19], biodegradation [20], and ion exchange [21]. These methods have been widely employed but are frequently insufficient for effective water treatment. While specific chemical and biological methods effectively remove dyes, most require significant energy consumption and complex technical procedures [22]. However, adsorption is one of the most important techniques used in several wastewater treatment domains due to its low production costs, low pollution and renewable biosorbent [23]. Finding a new, low-cost, highly effective biosorbent with remarkable adsorption characteristics throughout a wide pH range and the capacity to remove several organic dyes simultaneously is critical.

Biosorbents, which are adsorbents of biological origin, have high biosorption capacity, good adsorption kinetics, proper physical properties (size, shape, etc.), cost-effectiveness, easy availability, easy separation of the biosorbents from the solution, good thermal stability and chemical resistance, strong mechanical properties, and regeneration and reusability [24]. Many plant species have been used as biosorbents to decontaminate wastewater from heavy metals, dyes, etc [25, 26]. Recently, Akl et al. studied biogenic nano-silver doped grapefruit peels (GFP@ Ag) biocomposite for adsorptive photocatalytic degradation of organic pollutants [27]. Different biosorbents have been used for the removal of cationic and anionic dyes, such as banana peels [28], chestnut shells [29], palm kernel [30], pumpkin seed shells treated with sodium hydroxide [31], and nano-silver-doped flax fibers (NAgDFF) [32].

Corn kernels (CK) are small, edible seeds derived from the corn plant. Corn kernels are mainly composed of starch (62%), zein maize protein (19%), as shown in Fig. S1, water (15%), and oil (4%). Corn kernels may contain traces of other constituents, but these are small relative to the main components [33].

Previous studies have explored the use of various unmodified parts of the corn plant, such as corn cobs [34], corn husks [35], corn stalks [36], and corn silk [37] as natural biosorbents for the removal of pollutants from wastewater. However, CKs remain largely unexplored despite their promising physicochemical properties. CK is rich in starch, proteins, and diverse functional groups (e.g., hydroxyl, amine), enabling efficient interactions with cationic and anionic dyes through electrostatic attraction, π–π interactions, hydrogen bonding, and pore diffusion. In addition, CK offers practical advantages over other corn-derived materials, including ease of collection, minimal need for pre-processing, and preparation without chemical activation, making it suitable for low-cost, scalable applications.

The following goals guided the application of the current study: i—Preparation and characterization of CK biosorbent using FTIR, BET, pH_PZC_, elemental analysis, TGA, and SEM; ii—Studying the removal of the anionic AG20 and the cationic CV dyes from aqueous solutions using CK biosorbent using batch and column modes; iii—Investigating the various experimental factors affecting the adsorption process such as biosorbent dose, pH, initial dye concentrations, and ionic strength. ; iv—Performing kinetic and adsorption isotherm studies to comprehend the adsorption mechanism and the maximum adsorption capacity of CK biosorbent; v—Statistical analysis using the error functions *viz.* chi-square statistic (χ2), mean square error (MSE), hybrid error and the sum of squares error (SSE) ; vi—Application of CK biosorbent for the removal of AG20 and CV dyes from real water samples; vii—Elucidation of the adsorption mechanism of AG20 and CV onto CK biosorbent; viii—Selective adsorption of dyes in the mixture of AG20 and CV using CK biosorbent, and ix. Evaluation of the toxicity and leaching of the developed environmentally friendly biosorbent in view of the possibility of its release to the aqueous environment.

## Experimental

### Materials and chemicals

Pristine CK is collected from corn bought from the local market in Mansoura, Dakahlia, Egypt. AG20 and CV dyes, Table 1, were obtained from Sigma-Aldrich and were used without purification. Distilled water was used to prepare all chemical solutions. Stock solution (1000 mg/L) of AG20 was prepared by dissolving 1 g of AG20 in one liter of distilled water, and to prepare CV stock solution (1000 mg/L), 1 g of CV was dissolved in one liter of distilled water. NaCl (≥ 99.0%), Na_2_CO_3_ (≥ 99.5%), CH_3_COONa (≥ 99.0%), NaHCO_3_ (≥ 99.7%), NaNO_3_ (≥ 99.0%), KCl (≥ 99.0%), and ethanol (99.0%) were all analytical reagent grades and were purchased from Sigma Aldrich. The pH of the AG20 and CV solutions, was adjusted using NaOH (0.1 M) and/or HCl (0.1 M).

**Table 1:** Structure of dyes

Dyes	Chemical formula	Chemical structure	Molecular weight	λ_max_	Molecular diameter
Acid Green 20 (AG20)	(C_22_H_16_N_6_Na_2_O_7_S_2_)		586.51 g/mol	638 nm	20–25 Å.
Crystal Violet (CV)	(C_25_H_30_N_3_Cl)		407.98 g/mol	590 nm	14 Å.

### Preparation of CK biosorbent

The collected corn kernels were washed with ethanol and distilled water to remove all dirt before being dried in an oven at 80 °C until a stable weight was achieved, ground with a mill, and sieved to a mesh size of 0.5–0.6 mm.

### Characterization

The surface area of the CK biosorbent was assessed using Brunauer–Emmett–Teller (BET) methodology (Size Analyzer (QUANTACHROME–NOVA 2000 Series). The CNH composition of CK, CK-AG20, and CK-CV was determined using a Costech ECS-4010 elemental analyzer. Surface morphology of CK, CK-AG20, and CK-CV was determined using Scanning electron microscope (SEM-Quanta FEG-250). The FTIR spectra (ranged from 400 to 1 to 4000 cm^− 1^) of pristine CK, CK-CV, and CK-AG20 were measured using a Nicolet FTIR spectrophotometer (Perkin-Elmer Co., USA). The thermal stability of pristine CK biosorbent was investigated using thermogravimetric analyzer (Perkin Elmer TGA 4000) in the temperature range of 30–800 °C. A Perkin-Elmer 550 spectrophotometer was used for determining the absorption spectra of CV and AG20 and the mixture of AG20 and CV over a range of 190–900 nm. The point of zero charge(pH_PZC_) for pristine CK biosorbent was calculated as follows: a 25 mL 0.01 M NaCl solution with a pH range (2–12) was mixed with 0.01 g of CK biosorbent, and was shaken for 48 h on an equilibrated shaker. 0.1 M HCl and 0.1 M NaOH were added to NaCl to change the pH. The final pH was noted after shaking, and the formula was used to determine ΔpH = pH_i_-pH_f_. To determine the pH_PZC_ value, ΔpH was plotted against the initial pH (pH_i_). The pH_PZC_ value, ΔpH = 0, is the point where the X-axis (ΔpH) intersects the ΔpH vs. pH_i_ curve [38].

### Adsorption and regeneration procedures

#### Batch adsorption studies

The measured maximum adsorption spectra for AG20 and CV are 638 nm and 590 nm, respectively. Batch adsorption experiments were performed by shaking a known amount (0.01 g) of CK biosorbent with 10 mL of AG20 solution (100 mg/L) and CV solution (100 mg/L) in 150 mL stoppered bottles; then keeping the bottles in a thermostatic chamber and shaking at a constant speed of 200 rpm at room temperature. After reaching equilibrium, the supernatant solution containing the remaining portion from each dye was measured at their respective λ_max_. Various parameters affecting the adsorption of AG20 and CV, were investigated such as pH (2–10), temperature (25–45 °C), dose of CK (0.01–0.05 g), contact time, and initial concentrations of dyes (5–340) mg/L and ionic strength. The removal percentage (R%) and adsorption capacity (q_e_, mg/g) of pollutants, were calculated using Eqs. (1) and (2) [39].

1 $$\:\text{R}\text{\%}=\frac{\left(\text{C}\text{i}-\text{C}\text{o}\right)}{\text{C}\text{i}}\times\:100$$

2 $$\:\text{q}\text{e}=\frac{\begin{array}{c}\left(\text{C}\text{i}-\text{C}\text{o}\right)V\:\\\:\:\end{array}}{\text{m}}$$

where C_i_ (mg/L) and C_o_ (mg/L) are the initial concentration of dye and the concentration of dye at equilibrium after adsorption, respectively. m is the mass of the CK biosorbent (g), V (L) is the volume of the dye solution, and q_e_ is the amount of dye adsorbed (mg/g).

#### Desorption and regeneration studies

The desorption of dyes was examined using various kinds of eluents such as ethanol (99%), NaOH (0.1 M), Na_2_CO_3_ (0.1 M), CH_3_COONa (0.1 M), and HCl (0.1 M), following the adsorption of CV and AG20 by CK biosorbent. The regeneration process of CK biosorbent was studied using the batch method in five repeated cycles of adsorption-desorption. After shaking 0.01 g of CK biosorbent with 10 mL of 100 mg/L of each of CV and AG20 for two hours, the biosorbent was filtered and eluted with absolute ethanol. These steps were repeated four times more. The desorption efficiency (D% ) of the examined dyes was calculated using Eq. (3) [32, 40].

3 $$\:\:\:\text{D}\text{e}\text{s}\text{o}\text{r}\text{p}\text{t}\text{i}\text{o}\text{n}\:\text{e}\text{f}\text{f}\text{i}\text{c}\text{i}\text{e}\text{n}\text{c}\text{y}\:\left(\text{\%}\right)=\frac{{\text{C}}_{\text{d}\text{e}\text{s}}}{{\text{C}}_{\text{A}d\text{s}}}\times\:100\:\:\:\:\:\:\:\:\:$$

where C_des_ (mg/L) is the concentration desorbed into solution, and C_ads_ (mg/L) is the concentration originally adsorbed on sorbent.

#### Dynamic studies

A compressed mass of CK biosorbent was used to fill the bottom of the column. The flow rates of anionic and cationic dyes (slow 0.27 mL/min, 0.36 mL/min, and rapid 0.45 mL/min) were investigated using 100 mg/L of AG20 and CV at the optimum pH obtained from the batch experiment. The optimal parameters were column diameter (0.3 cm, 0.7 cm, and 0.9 cm) and the CK weight (0.005 g to 0.03 g). The remaining concentrations of AG20 and CV were spectrophotometrically measured at their respective λ_max_ after passing the feed solutions through the column. The removal percentage (R%) was computed using Eq. (1).

#### Effect of initial dye concentration and isotherm studies

The isotherm studies for the adsorption of AG20 and CV were carried out by inserting 0.01 g of CK biosorbent in several bottles containing solutions of AG20 and CV dyes. The initial concentration of each dye was between 5 and 340 mg/L. These bottles were shaken in a thermostatic shaker for 120 min, at 150 rpm at 25 °C for CV at pH 8 and at pH 3 for AG20. The linear and nonlinear forms of the Langmuir, Freundlich, and the linear form of Dubinin–Radushkevich (D–R) isotherm models were used. The parameters were determined using Eqs. (4) and (5) for the linear form of the Langmuir and the Freundlich isotherm models, respectively, and Eqs. (6) and (7) for nonlinear forms of the Langmuir and the Freundlich isotherm models, respectively. The parameters of D–R isotherm were calculated using Eq. (8) [41–44].

4 $$\:\frac{Co}{qe}=\frac{1}{{K}_{L}{q}_{m}}+\frac{Co}{{q}_{m}}$$

5 $$\:{ln}{q}_{e}={ln}k+\frac{1}{n}In{C}_{e}$$

6 $$\:{q}_{e}=\frac{{c}_{e}\times\:{K}_{L}\times\:{q}_{m}}{{c}_{e}\times\:{K}_{L}+1}$$

7 $$\:{q}_{e}={C}_{e}^{\frac{1}{n}}\times\:{K}_{F}$$

8 $$\:\:{ln}{q}_{e}={ln}{q}_{m}-{K}_{D}\:{\epsilon\:}^{2}$$

where C_o_ (mg/L) is the concentration of AG20 and cationic CV at equilibrium, q_e_ (mg/g) is the adsorption capacity of dye at equilibrium, q_m_ (mg/g) is the maximum amount of adsorption, 1/n is the heterogeneity factor, K_D_ (mol²/kJ²), K_L_ (L/mg), and K_F_ (mg/g) (L/mg)^1/n^ are the Langmuir, Freundlich, and D–R isotherm related to adsorption energy, and $\:{{\upepsilon\:}}^{2}$Polanyi potential, respectively.

The separation parameter, R_L_, shown in Eq. (9), is a critical dimensionless factor that is utilized in the prognosis of adsorbate-adsorbent affinity. The R_L_ value provides important insights into the adsorption process’s favorability. If it is (R_L_ ˃ 1), the studied adsorbent is unsuitable and unfavorable. On condition that it is (0 < R_L_ < 1), (R_L_ = 0), or (R_L_ = 1), the reaction is suitable with high affinity towards adsorbate, irreversible, or linear, respectively [45].

9 $$\:{R}_{L}=\frac{1}{1+{k}_{L}{C}_{0}}$$

#### Effect of contact time and kinetic studies

Kinetic studies were conducted using two kinetic models, pseudo-1st order (PFO), pseudo-2nd order (PSO), and intraparticle diffusion (IPD) in order to determine the adsorption rate-limiting steps and enable a detailed assessment of the adsorption mechanism. The parameters of the linear form of PFO and PSO are calculated using Eqs. (10), (11), (12). Equation (13) and Eq. (14) are used to calculate the parameters of the nonlinear and linear forms of IPD, respectively [46–49]. The investigations were carried out at the optimum pH for each dye using 10 mL (100 mg/L of each of AG20 and CV ) and 0.01 g of the CK biosorbent.The contact times ranged from 5 to 360 min.

10 $$\:\frac{1}{{q}_{t}}=\frac{{k}_{1}}{{q}_{e1}t}+\frac{1}{{q}_{e1}}$$

11 $$\:\frac{t}{{q}_{t}}=\frac{1}{{k}_{2}{q}_{e2}^{2}}+\frac{t}{{q}_{e2}}\:\:\:$$

12 $$\:{q}_{t}={q}_{e1}\left(1-{e}^{-{k}_{1}t}\right)\:\:\:$$

13 $$\:{\text{q}}_{\text{t}}=\frac{{\text{q}}_{\text{e}1}^{2}{\text{k}}_{2}\text{t}}{{\text{q}}_{\text{e}1}{\text{k}}_{2}\text{t}+1}\:\:\:\:\:\:\:\:\:$$

14 $$\:{\text{q}}_{\text{t}}={\text{k}}_{\text{d}\text{i}\text{f}\text{f}}{\text{t}}^{\raisebox{1ex}{$1$}\!\left/\:\!\raisebox{-1ex}{$2$}\right.}+1\:\:\:\:\:\:\:\:$$

where q_e_ (mg/g) and q_t_ (mg/g) are the adsorption capacity of for the dyes at equilibrium and at a specific time t (min), respectively. k_1_ and k_2_ are rate constants for pseudo-1st and pseudo-2nd order, and k_diff_ is the IPD rate constant.

#### Estimation of the error functions

The well-fitted kinetic and isotherm models were examined using a variety of error functions which helped to reduce the error distribution between the values derived from theoretical model correlations and experimental data. Four error functions were used viz. the sum of squares error (SSE), mean square error (MSE), hybrid error, and chi-square statistic (χ2), Eqs. (15), (16), (17), (18) [50–52].

15 $$\:\:{\text{x}}^{2}=\:\:\sum\:_{\text{i}=1}^{\text{n}}{\frac{\:\:\left(\:{\text{q}}_{{\text{e}}_{\text{i}}^{\text{e}}}\text{x}\text{p}-{\text{q}}_{{\text{e}}_{\text{i}}}\text{c}\text{a}\text{l}\right)}{{\text{q}}_{\text{e}\text{i}}\text{c}\text{a}\text{l}}}^{2}\:\:\:$$

16 $$\:\:\text{M}\text{S}\text{E}=\frac{1}{\text{N}\text{e}\text{x}\text{p}}\:\sum\:_{\text{i}=1}^{\text{n}}{\left(\:{\text{q}}_{{\text{e}}_{\text{i}}^{\text{e}}}\text{x}\text{p}-{\text{q}}_{{\text{e}}_{\text{i}}}\text{c}\text{a}\text{l}\right)}^{2}\:\:$$

17 $$\:\:\text{S}\text{S}\text{E}=\:{\sum\:}_{\text{i}=1}^{\text{n}}{\:\left(\:{\text{q}}_{{\text{e}}_{\text{i}}^{\text{e}\text{x}\text{p}}}-{\text{q}}_{{\text{e}}_{\text{i}}}\text{c}\text{a}\text{l}\right)}^{2}\:\:\:\:\:\:\:\:\:\:\:\:\:\:\:\:\:\:$$

18 $$\:\text{H}\text{y}\text{b}\text{r}\text{i}\text{d}\text{e}\:\text{e}\text{r}\text{r}\text{o}\text{r}=\frac{100}{\text{N}\text{e}\text{x}\text{p}-\text{N}\text{p}\text{a}\text{r}\text{a}\text{m}\text{e}\text{t}\text{e}\text{r}}\:\sum\:_{\text{i}=1}^{\text{n}}{\frac{\:\:\left({\text{q}}_{{\text{e}}_{\text{i}}^{\text{e}}}\text{x}\text{p}-{\text{q}}_{{\text{e}}_{\text{i}}}\text{c}\text{a}\text{l}\right)}{{\text{q}}_{\text{e}\text{i}}\text{e}\text{x}\text{p}}}^{2}\:\:$$

where n is the number of included observations. The subscript *cal* refers to theoretically calculated data, while the *exp* subscript represents experimental data.

#### Effect of temperature and thermodynamic studies

A series of 100 mL stoppered bottles containing 10 mL of AG20) 100 mg/L (and CV) 100 mg/L ( and 0.01 g of CK biosorbent at varying temperatures (298–323 °K), and pH 3 for AG20 and pH 8 for CV were shaken for 120 min using an equilibrated shaker at a constant speed of 150 rpm. After adsorption and filtration, the concentrations of residual AG20 and CV dyes were determined.

The thermodynamic parameters: adsorption enthalpy (ΔH°), adsorption free energy (ΔG°), and adsorption entropy (ΔS°) are determined using Eqs. (19), and (20) [53, 54].

19 $$\Delta {\rm{G}}^\circ = - {\rm{RT}}\ln {{\rm{K}}_{\rm{c}}}$$

20 $${\text{ln}}{{\text{K}}_{\text{C}}}{\text{=}}\frac{{{\text{\varvec{\Delta}}}{{\text{S}}^{\text{o}}}}}{{\text{R}}} - \frac{{{\text{\varvec{\Delta}}}{{\text{H}}^{\text{o}}}}}{{{\text{RT}}}}$$

The values of ΔS° and ΔH° were determined using Eq. (20), where the intercept equals ΔS°/R and the slope equals − ΔH°/R of Ln Kc vs. 1/T. A gas constant (R) value is 8.314 J/mol K [55].

#### pH-responsive removal of AG20 and CV in a mixture

0.01 g of CK biosorbent was added to a binary dye solution (100 mg/L of each dye) at pH 3 and 8, followed by shaking at 120 rpm for 2 h. The concentration of dye at equilibrium was determined using UV–Vis spectrophotometry, and the removal efficiency was calculated according to Eq. (1).

#### Application

Three different water samples (tap water, sea water and wastewater) were utilized to investigate and evaluate the applicability of the prepared CK biosorbent. The organic matters of the the investigated water samples were digested as follows: a mixture composed of 0.5 g K_2_S_2_O_8_ and 5 mL of H_2_SO_4_ (95.0–98.0% (w/w)) was added to each water sample, followed by heating at 90 °C for 2 h. An amount of (50&100) mg/L of each AG20 and CV was spiked into each of the digested samples. Then, the spiked samples were mixed with 0.01 g of CK biosorbent, and the pH value was adjusted to 3 for AG20 and to 8 for CV with continuous shaking for 2 h. After that, the solutions were centrifuged, and another 0.01 g of CK biosorbent was added to the supernatant and the previous steps were repeated to ensure the complete separation of dyes. Finally, the remaining amounts of AG 20 and CV in the supernatant solution were determined spectrophotometrically at the appropriate wavelengths.

### Toxicity and leaching studies of CK before and after dye binding (CV- and AG20-CK)

Two types of bacteria: Gm + ve bacteria (*S. aureus*) and Gm-ve bacteria (*E. coli*) were used to examine the toxic effects of CK before and after dye binding (CV- and AG20-CK) using the agar well diffusion method [56]. Similar to the procedure used in the disk-diffusion method, the agar plate surface is inoculated by spreading a volume of the microbial inoculum over the entire agar surface. Then, a hole with a diameter of 9 mm is punched aseptically with a sterile cork borer or a tip, and a volume (100 µL) of each sample, which is suspended in two types of solvent (water and DMSO) in the desired concentration (35 mg/L), is introduced into the well. The samples were seeded in Petri dishes containing agar media and were incubated for five h at 36 °C. The toxicity was recorded by measuring the diameter of the zone of inhibition after 24 h of incubation. The obtained data were compared with the standard Amoxicillin drug [56].

The water solubility was used to examine the leaching of CK before and after dye binding (CV- and AG20-CK). 1 g of each CK, CV-CK, CK, and AG20-CK was suspended in 50.0 mL of DDW to determine the substance’s solubility in water. The amount of each sample that resulted from stirring the suspension for about 3.0 h was filtered out and dried [56].

## Results and discussion

### Adsorption efficiency of different parts of corn

Preliminary experiments were performed to study the removal efficiency of the different parts of corn viz. Corn Kernel (CK), corn husk (CH), Corn Pedicle (CP), Corn Silk (CS), and Corn Cob (CC) towards the removal of AG20 and CV from aqueous solution at the optimum condition. The results are presented in Fig.S2 and tabulated in Table 2. Upon comparing the results obtained, it can be noticed that CK has the highest the removal % of the CV and AG20.

**Table 2:** Removal percent of AG20 and CV by the different parts of corn

Corn parts	Dye (100 mg/L)	Removal percentage (*R*,% )
Corn husk (CH)	AG20	27.8
	CV	57.3
Corn pedicel (CP)	AG20	29.02
	CV	54.62
Corn kernel (CK)	AG20	85.74
	CV	61.1
Corn silk (CS)	AG20	41.3
	CV	50.07
Corn cob (CC)	AG20	24.6
	CV	38.95

### Physicochemical studies

#### Optical images

**Fig. 1:**
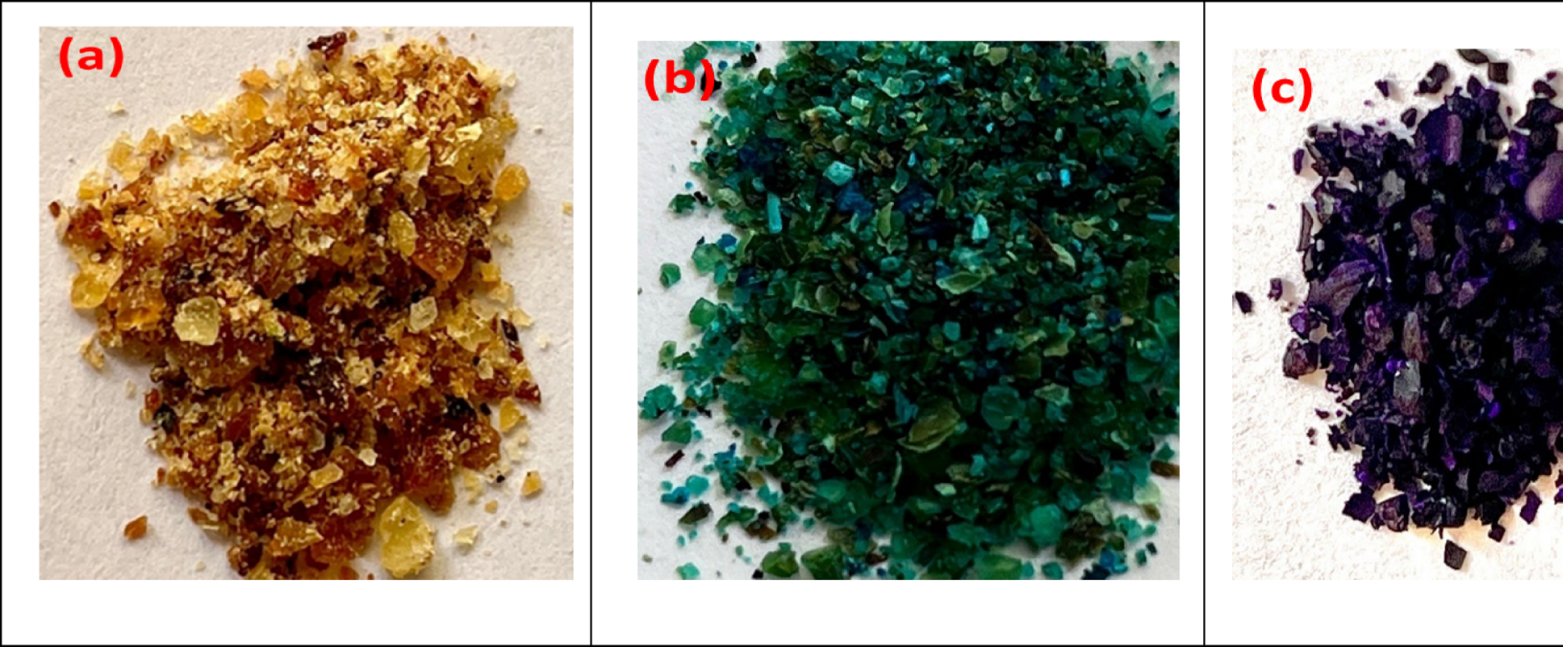
Digital photographs of **a** pristine CK, **b** CK-AG20, **c** CK-CV

The optical images of pristine CK, CK-AG20, and CK-CV are shown in Fig. 1a–c. As it can clearly be noticed, the color of the pristine CK biosorbent changed from golden yellow (Fig. 1a) to green for CK-AG20 (Fig. 1b) and violet for CK-CV (Fig. 1c), after adsorption, respectively.

#### BET

The surface area and textural characteristics of the CK biosorbent were determined using the BET method. Nitrogen adsorption–desorption measurements were conducted at 77.35 K, and the adsorption data were analyzed using the linear BET equation. The BET surface area was calculated from the linear region of the BET plot, as shown in Fig.S3. As can be noticed from Table 3, the average pore diameter of the CK biosorbent is 46.67 Å, which is greater than the molecular diameter of AG20 (20–25Å) and the molecular diameter of CV 14Å. This would allow the feasible pore diffusion of dyes into the CK biosorbent.

**Table 3:** The textural properties of CK biosorbent

Biosorbent	Adsorption Temp.(K)	BET C constant	Aver. pore diameter (Å)	Total pore volume (cm^3^.g^− 1^)	Surface area (m^2^.g^− 1^)
CK	77.35	24.886	46.672	0.1200	51.4286

### Characterization

#### Elemental analysis

The elemental composition of pristine CK, CK–AG20, and CK–CV was analyzed, and the results are presented in Table 4. Sulfur was detected only in the CK–AG20 sample (0.58%), confirming the successful adsorption of AG20 (C_22_H_16_N_6_Na_2_O_7_S_2_) on to CK biosorbent. The carbon percentage increased from 39.2% in CK to 40.9% in CK–CV and 42.1% in CK–AG20. Similarly, the hydrogen content increased from 5.9% in CK to 6.01% in CK-CV and to 6.2% in CK-AG20.

**Table 4:** Elemental analysis of CK, CK-AG20, and CK-CV

Sample	C(%)	H(%)	*N*(%)	S(%)
CK	39.2	5.9	1.7	0
CK-AG20	42.1	6.2	2.2	0.58
CK-CV	40.9	6.01	1.5	0

#### SEM

**Fig. 2:**
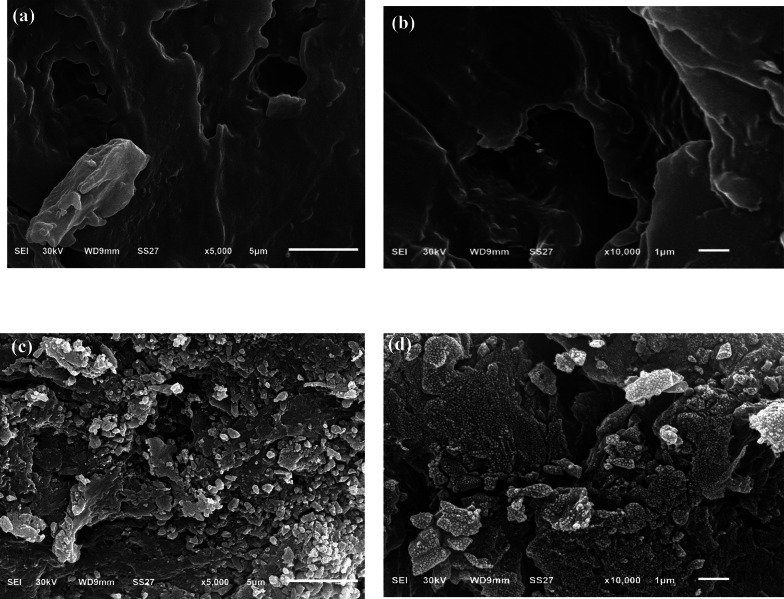
SEM images of a,b CK biosorbent, c CK-CV and d CK-AG20

The SEM images presented in Fig. 2a–d reveal the surface morphology of CK biosorbent before and after adsorption of AG20 and CV dyes. The surface morphology of pristine pristine CK (Fig. 2a, b) exhibits a heterogeneous, rough morphology with visible pores and irregular channels. Compared to the SEM image of pristine CK, the SEM image of CK-CV (Fig. 2C) appears smoother and more compact, with a noticeable reduction in the number of visible pores. This suggests that CV molecules have filled the surface pores or covered the active sites of CK biosorbent, indicating successful adsorption of CV onto CK biosorbent [57, 58]. The SEM image of CK-AG20 (Fig. 2d), displays an almost entirely covered texture, with minimal visible pores. This dense layer indicates that a substantial amount of AG20 molecules adhered to the surface, reflecting the high adsorption capacity of CK biosorbent.

#### FTIR analysis

**Fig. 3:**
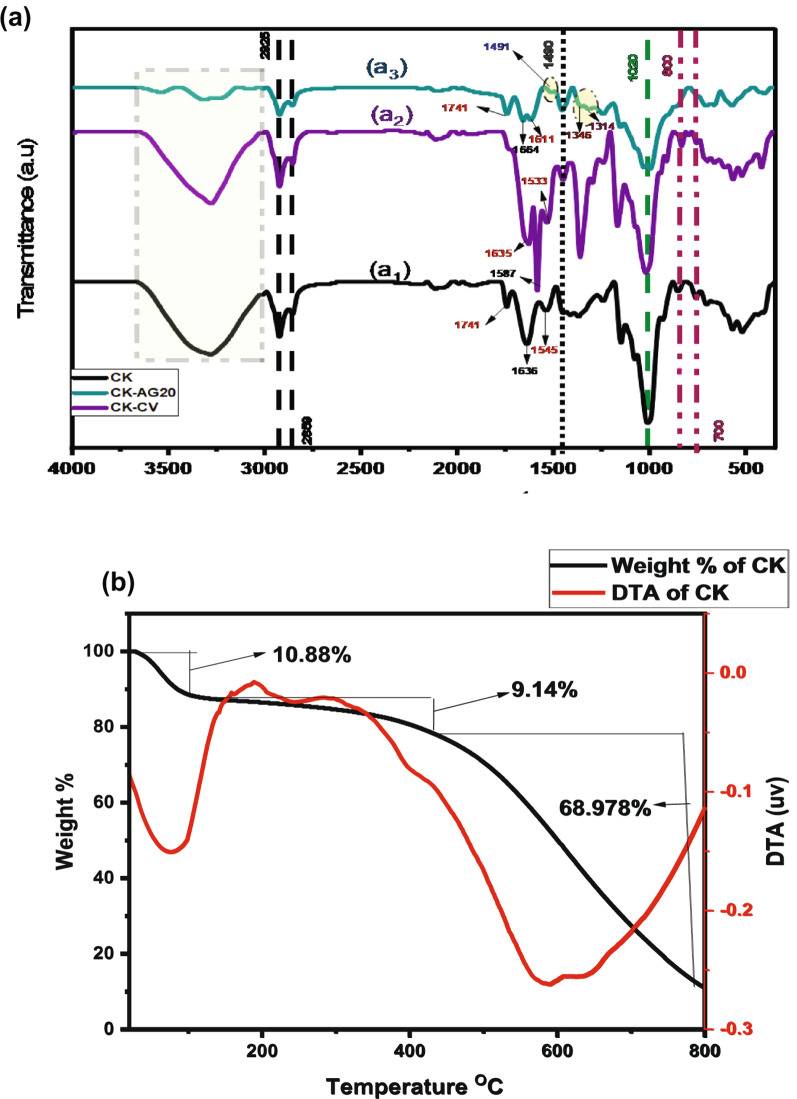
**a** FTIR spectra of: a_1_ CK, a_2_ CK-CV, and a_3_ CK-AG20, and **b** thermal analysis of CK biosorbent

The FTIR spectra of pristine CK, CK-CV, and CK-AG20, presented in Fig. 3(a_1_–a_3_), were investigated in the 400–4000 cm⁻¹ to examine the functional groups involved in dye adsorption. A summary of the peaks of FTIR spectra and their corresponding functional groups is provided in Table S1.

The FTIR spectrum of pristine CK (Fig. 3a_1_) exhibits several characteristic peaks corresponding to its main components, primarily starch and zein maize proteins. A broad absorption band between 3400 cm⁻¹ and 3200 cm⁻¹ is attributed to O-H stretching vibrations of hydroxyl groups and overlapping contributions from carboxylic acid (–COOH) groups involved in hydrogen bonding. The bands around 2925 cm⁻¹ and 2859 cm⁻¹ correspond to C–H stretching in –CH₃ and –CH₂ groups which are characteristic of the aliphatic chains in starch and zein proteins [58, 59]. An absorption band at 1741 cm⁻¹ is assigned to C=O stretching of carbonyl groups. Two prominent peaks at 1636 cm⁻¹ and 1545 cm⁻¹ are associated with amide I (C=O stretch) and amide II (N–H bending + C–N stretch), respectively, confirming the presence of zein protein. Additional peaks below 1400 cm⁻¹ indicate various peptide conformations. A peak at 1490 cm⁻¹ corresponds to aromatic C=C stretching, especially from phenyl alanine residues. A 1050–1030 cm⁻¹ band indicates C–O–C stretching from glycosidic linkages in starch.

The FTIR spectrum of CK-CV (Fig. 3a_2_) shows distinct shifts of the amide I band at 1636 cm⁻¹ to 1587 cm⁻¹, and the carbonyl peak at 1741  to 1635 cm⁻¹, indicating strong interactions between CV molecules and surface functional groups of CK biosorbent. Additionally, the amide II peak at 1545 cm⁻¹ ( in FTIR spectrum of CK) becomes less intense, suggesting potential dye binding to amino groups. The band at 926 cm⁻¹ shifts to 914 cm⁻¹, indicating structural rearrangement upon dye adsorption.

In the FTIR spectrum of CK-AG20 (Fig. 3a_3_), the amide II and I bands shift to 1611 cm⁻¹ and 1664 cm⁻¹, respectively. New doublet peaks appear at 1314 cm⁻¹ and 1346 cm⁻¹ corresponding to symmetric and asymmetric sulfate (SO₃²⁻) stretching. This would suggest the interaction with sulfonic groups in AG20 dye [60]. The skeletal band at 926 cm⁻¹ shifts to 900 cm⁻¹, reflecting additional molecular interactions or structural changes.

Overall, the FTIR spectra demonstrate significant structural and chemical modifications, including peak shifts, new band appearances, and intensity changes, confirming the successful adsorption of CV and AG20 onto CK biosorbent.

#### TGA

The thermal stability of CK biosorbent was estimated using TGA (Fig. 3b) in the temperature range of 25–800 °C by recording weight loss as temperature increased up to 800 °C. The CK biosorbent decomposes in three steps. The first step shows a weight loss of 10.88% at temperatures ranging from 25 to 170 °C. This generally corresponds to water evaporation. In the second step, a weight loss of 9.14% occurred at temperature ranging from 170 to 450 °C. This could be due to thermal degradation of low molecular weight organic compounds such as zein protein and simple carbohydrates. In the third step, a weight loss of 68.98% developed at temperatures ranging from 450 to 800 °C. This can be due to degradation of the starch [61].

### Adsorption studies

#### Point of zero charge (pH_PZC_)

The changes in pH value (pH_initial__pH_final_) for CK as a function of pH_initial_ are shown in Fig. S4. From Fig. S4, the pH_pzc_ value of CK biosorbent is determined to be 5.9. This result indicates that at a pH below 5.9, the surface of the CK biosorbent displayed positive charges, while at a pH above 5.9, the surface displayed negative charges.

#### Effect of pH

**Fig. 4:**
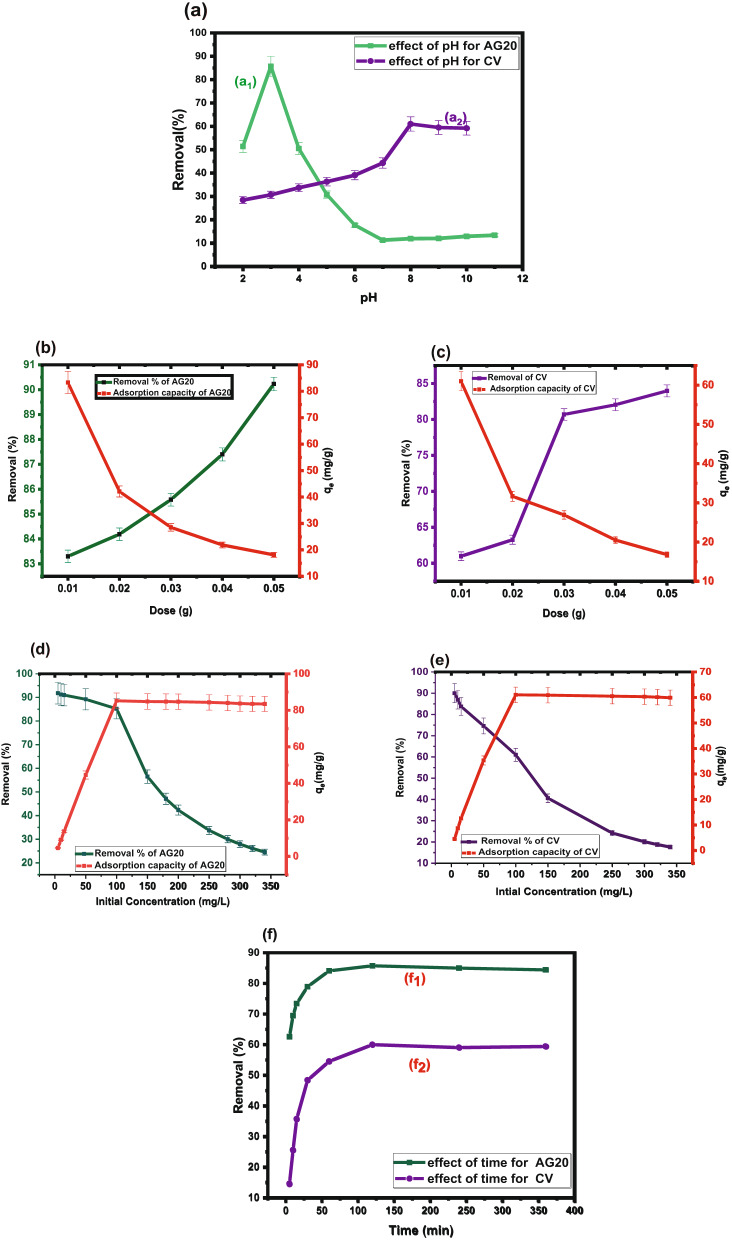
**a** Effect of pH on the adsorption of (a1) AG20 and (a2) CV on to CK biosorbent (conditions: temp. 25 °C, dose: 0.01 g, conc.: 100 mg/L, volume: 10 mL,120 min); **b** Effect of dose on the adsorption of AG20 (conditions: temp.: 25 °C, pH = 3 for AG20, conc.: 100 mg/L, volume: 10 mL, 120 min), **c** Effect of dose on the adsorption of CV (conditions; temp. 25 °C, pH = 8, conc.: 100 mg/L, volume: 10 mL,120 min), **d** Effect of initial conc. of AG 20 (pH = 3, temp.: 25 °C, dose: 0.01 g, volume: 10 mL, 120 min), **e** Effect of initial conc of CV (pH = 8, temp.: 25 °C, dose: 0.01 g, volume: 10 mL,120 min) adsorption on CK, **f** Effect of time on adsorption of f_1_ AG 20 (pH = 3, temp.: (25 °C), dose: 0.01 g, volume: 10 mL, 100 mg/L), and f_2_ CV (pH = 8, temp.: (25 °C), dose: 0.01 g, volume: 10 mL,100 mg/L) adsorption on CK

The effect of pH, Fig. 4a, is a critical factor influencing the adsorption efficiency of both dyes. The pH was varied from 2 to 10 to investigate its impact. As presented in Fig. 4a_1_, the removal of AG20 increased sharply, reaching a maximum of 85.74% at pH 3, then gradually decreases at higher pH values. In contrast, Fig. 4a_2_ shows that CV removal increased with the increase of pH, reaching a peak of 61.1% at pH 8, followed by a slight decline at more alkaline conditions.

These trends can be explained by the (pH_pzc_) of CK biosorbent. At At pH < 5.9, the surface of CK biosorbent is positively charged, favoring the adsorption of anionic dyes like AG20 due to electrostatic attraction. Conversely, at pH >5.9, the surface of CK becomes negatively charged enhancing the adsorption of the CV cationic dye. Therefore, the high removal % of AG20 at acidic pH = 3 is attributed to favorable electrostatic interactions, while the increased adsorption of CV at basic pH = 8 results from attraction between positively charged dye molecules and the negatively charged surface of CK biosorbent [62].

#### Effect of dose

The effect of the dose of CK biosorbent was examined using different amounts of CK biosorbent in 10 mL of 100 mg/L of AG20 and CV, as shown in Fig. 4b, c, respectively, with a shaking time of 120 min at room temperature. As shown in Fig. 4b, with the increase in CK dose from 0.01 to 0.05 g, the adsorption capacity of AG20 decreased from 85.74 to 18.1 mg/g, while in Fig. 4c, the adsorption capacity of CV decreased from 61.1 to 16.64 mg/g. This means that the available active sites on the CK biosorbent in solution are more than the number of dye molecules. As a result, many sites remain unoccupied, leading to a decrease in the adsorption capacity of CK biosorbent [63].

#### Effect of initial concentration of dyes and adsorption isotherms

The influence of initial dye concentration (5–360 mg/L) on the adsorption capacity of AG20 and CV, was evaluated. As shown in Fig. 4(d, e), the adsorption capacity of AG20 increased from 4.95 to 85.34 mg/g, while CV increased from 4.16 to 61.1 mg/g with increasing the initial concentration of dyes to 100 mg/L. Beyond this point, the adsorption capacity attained a plateau, indicating that the active binding sites on the surface of CK became fully saturated, and the adsorption equilibrium is reached.

Various isotherm models viz. the linear and nonlinear forms of Langmuir and Freundlich, and the linear form of Dubinin–Radushkevich (D–R) are used to interpret the adsorption equilibrium. Fig. S5(a–e) shows the determined Langmuir and Freundlich isotherms for the adsorption of AG20 and CV utilizing CK biosorbent and their derived parameters (K_L_, K_f_, n, and q_m_) are presented in Table 5. From the results in Table 5, it can be noticed that the adsorption process of AG20 and CV utilizing CK biosorbent follows the Langmuir isotherm model as it has a higher R^2^ value and lower χ^2^, SSE, MSE, and hybrid error function values compared to those of the Freundlich model. The R_L_ values were estimated to be less than 1.0, which signifies that the adsorption of AG20 and CV utilizing CK biosorbent is favorable and demonstrates its applicability for the remediation of these dyes from various solutions.

Regarding the nonlinear models, the nonlinear Langmuir model predicted slightly higher q_m_ values (86.97 mg/g for AG20 and 72.29 mg/g for CV) with relatively high R^2^ values of 0.9483 for AG20 and 0.9540, respectively. Although these values indicate good fitting, they were still less than those obtained using the linear Langmuir form. The nonlinear Freundlich model showed lower R^2^ values of 0.8389 for AG20 and 0.850 for CV. These findings confirm that the linear Langmuir model remains the most appropriate isotherm model for adsorption of AG2 and CV on CK biosorbent.

The Dubinin–Radushkevich (D-R) isotherm model was utilized to estimate the adsorption energy (E) and determine the nature of the adsorption (Table 5). The adsorption energy (E) value provides details about the physical or chemical nature of the adsorption process. When E is less than 8 kJ/mol, physisorption can explain the type of adsorption; when E is greater than 8 kJ/mol, ion-exchange or chemical adsorption can control it. The D-R isotherm fitting curve is displayed in Figure S5e, and Table 5 contains the determined parameters. The calculated adsorption energy (E < 8 kJ/mol) indicates that physisorption can account for the type of adsorption of studied dyes onto CK biosorbent. This means that adsorption is reversible and is characterized by the formation of weak physical attraction forces between adsorbate molecules and the solid surface, such as electrostatic attraction, and Van der Waals forces [64].

**Table 5:** Adsorption isotherm parameters of AG20 and CV by CK

Model	Parameters	CK-AG20	CK-CV
Linear Langmuir	q_m_ (mg/g)	85.69	61.99
	K_L_(min^− 1^)	0.242	0.144
	R^2^	0.9993	0.99910
	R_L_	0.0397	0.065
	χ2	225.62	150.315
	SSE	19333.62	9319.54
	MSE	1487.202	931.95
	Hybrid error	20763.28	14182.84
Linear Freundlich	n	12.14	8.37
	K_F_(min^− 1^)	0.4011	0.3950
	R^2^	0.8483	0.9110
	χ2	653.83	167.052
	SSE	23407.14	5938.70
	MSE	1800.55	593.87
	Hybrid error	4803.14	4454.19
Nonlinear Langmuir	q_m_ (mg/g)	86.98	72.29
	K_L_(min^− 1^)	0.0215	0.022
	R^2^	0.9483	0.954
Nonlinear Freundlich	q_m_ (mg/g)	9.0314	7.368
	K_F_ (min^− 1^)	0.37730	0.379
	R^2^	0.8390	0.850
Linear D-R	q_m_ (mg/g)	60.58	44.67
	E (kJ\mol)	1.085	1.183
	R^2^	0.8099	0.7053

#### Effect of oscillation time and kinetic studies

The contact time is a key parameter controlling the adsorption process. As shown in Fig. 4f1, for AG20, the removal efficiency increased rapidly from 62.6% at 5 min to 85.74% at 120 min, after which no significant change was observed indicating that equilibrium was attained at 120 min. A similar trend was observed for CV, Fig. 4g, where the removal efficiency increased from 14.6% at 5 min to 61.05% at 120 min. Above 120 min the curve attained a plateau indicating that equilibrium is reached at 120 min.

To evaluate the adsorption kinetics, both linear and nonlinear forms of the pseudo-first-order (PFO) and pseudo-second-order (PSO) models were applied, along with the linear form of the intra-particle diffusion (IPD) model, Eqs. (10–14). The kinetic parameters, derived from the slopes and intercepts of Fig.S6(a–e), including correlation coefficients (R²) and constants (k₁, k₂, kdiff, ${{\rm{q}}_{{{\rm{e}}^{\rm{1}}}}}$, qdiff, ${{\rm{q}}_{{{\rm{e}}^{\rm{2}}}}}$), are presented in Table 6.

A comparative analysis between the linear and nonlinear forms of the PFO and PSO models revealed that the PFO model exhibited limited predictive reliability. In its linear form, the CK–AG20 system yielded R² = 0.9746 with ${{\rm{q}}_{{{\rm{e}}^{\rm{1}}}}}$ = 44.52 mg/g, and the CK–CV system gave R² = 0.9849 with ${{\rm{q}}_{{{\rm{e}}^{\rm{1}}}}}$ = 3.713 mg/g. The nonlinear PFO form improved ${{\rm{q}}_{{{\rm{e}}^{\rm{1}}}}}$ estimates (82.09 mg/g for CK–AG20 and 56.86 mg/g for CK–CV) but still showed lower R² values (0.97701 and 0.98134, respectively) compared to PSO. These deviations from experimental q_e_ values indicate that PFO is less suitable for describing the adsorption kinetics.

In contrast, the PSO model provided a better fit. The linear PSO form yielded R² values of 0.9998 and 0.9989 for CK–AG20 and CK–CV, respectively; with the calculated ${{\rm{q}}_{{{\rm{e}}^{\rm{2}}}}}$ values (84.32 and 61.31 mg/g) closely matching experimental results.

For, the nonlinear PSO form the R² and ${{\rm{q}}_{{{\rm{e}}^{\rm{2}}}}}$ for CK–AG20 are 0.9973 and 85.56 mg/g, resectively, while the R² and ${{\rm{q}}_{{{\rm{e}}^{\rm{2}}}}}$ for CK-CV are 0.9901 and 63.45 mg/g, respectively.

In conclusion, the correlation coefficients (R^2^ = 0.999) corresponding to the PSO for both studied dyes are larger than those for the PFO. Furthermore, analysis of the data of error functions revealed that the the PSO model has lower values of error functions compared to the PFS. These findings demonstrate that PSO, particularly in its linear form, best describes the adsorption kinetics of both dyes onto CK biosorbent.

The intra-particle diffusion (IPD) model was applied to elucidate the adsorption mechanism. As shown in Fig.S6e, the plots exhibited a clear multi-linear pattern with three distinct regions, each representing a specific adsorption stage. The initial steep segment corresponds to the external mass transfer (film diffusion) phase, where dye molecules migrate rapidly from the bulk solution to the biosorbent surface. The second linear portion is attributed to intra-particle diffusion through the pores of the biosorbent. At the same time, the final plateau region represents the equilibrium stage, where the adsorption rate declines as the available active sites become saturated. None of the IPD lines intersected the origin, indicating that intra-particle diffusion is not the only rate-controlling step. Instead, film diffusion and boundary layer resistance play significant roles in governing the adsorption process [65].

**Table 6:** Kinetic parameters for the AG20 and CV adsorption

System	Parameters	CK-AG20	CK-CV
Linear PFO model	${{\rm{q}}_{{{\rm{e}}^{\rm{1}}}}}$ (mg/g)	44.52	3.713
	k_1_ (min^− 1^)	1.91	0.0512
	R^2^	0.9746	0.9849
	χ2	212.48	1941.94
	SSE	9459.21	15535.48
	MSE	1182.40	4184.08
	Hybrid error	8.19	21.05
Linear PSO model	${{\rm{q}}_{{{\rm{e}}^{\rm{2}}}}}$ (mg/g)	84.32	61.31
	k_2_ (min^− 1^)	0.024	0.0016
	R^2^	0.99980	0.9989
	χ2	9.990	70.713
	SSE	842.51	4335.40
	MSE	105.30	541.93
	Hybrid error	0.890	15.53
Nonlinear PFO model	${{\rm{q}}_{{{\rm{e}}^{\rm{1}}}}}$ (mg/g)	82.09	56.86
	k_1_ (min^− 1^)	0.240	0.059
	R^2^	0.9770	0.9810
Nonlinear PSO model	${{\rm{q}}_{{{\rm{e}}^{\rm{2}}}}}$ (mg/g)	85.56	63.45
	k_2_ (min^− 1^)	0.0057	0.0012
	R^2^	0.99730	0.9901
Linear IPD	q_e_ (mg/g)	81.21	56.76
	k_diff_ (mg.g^− 1^.min^− 1^)	1.214	2.519
	R^2^	0.655	0.689

#### Thermodynamic studies

The thermodynamic parameters (Gibbs free energy change (ΔG°), enthalpy change (ΔH°), and entropy change (ΔS°) were determined to understand the nature of adsorption of AG20 and CV on the CK biosorbent, over a temperature range of 298–323 K. These parameters, along with the equilibrium constant (Kc), were calculated using Eqs. (19), and (20). Table 7 displays the estimated thermodynamic values, while Fig.S7(a-b) shows the corresponding linear plots of ln Kc versus 1/T for AG20 and CV, respectively.

The negative ΔG° values for both dyes across the studied temperature range suggest that the adsorption process is spontaneous. Additionally, the positive ΔH° values indicate that the adsorption is endothermic. The positive ΔS° values also suggest increased randomness at the solid-liquid interface during adsorption, reflecting a higher degree of molecular disorder [66].

The linearity of the ln Kc versus 1/T plots across the temperature range further supports the reliability of the calculated thermodynamic parameters. It confirms the consistent temperature dependence of the adsorption behavior, as shown in Fig. S7.

**Table 7:** Thermodynamic parameters for the adsorption of AG20 and CV onto CK biosorbent

System	T(^o^K)	K_C_	∆G^o^ (kJ/mol)	∆H^o^_ads_ (kJ/mol)	∆S^o^ (J/mol.K)
CK-AG20	298	6.01	− 4.43	33.13	134.19
	308	9.36	− 5.73		
	323	21.32	− 8.216		
CK-CV	298	1.6	− 1.164	48.399	168.36
	308	5.1	− 4.172		
	323	9.18	− 5.954		

#### Effect of ionic strength

The ionic strength is a crucial parameter because industrial wastewater contains significant concentrations of various solutes. The ionic strength was examined utilizing several anions such as NaCl (0.1 M), Na_2_CO_3_ (0.1 M), CH_3_COONa (0.1 M), NaHCO_3_ (0.1 M), NaNO_3_ (0.1 M), and KCl (0.1 M) at the optimal conditions of the adsorption process. Fig.S8a illustrates the effect of various anions on the adsorption of AG20 and CV on to CK biosorbent. NaCl (0.1 M) significantly affected the AG20, while Na_2_CO_3_ (0.1 M) considerably influenced the adsorption of CV. The influence of NaCl on the removal % of AG20 and the influence of Na_2_CO_3_ on the removal % of CV using CK biosorbent, are shown in Fig. S8b. For AG20, the removal % increased from 85.74 to 90.87 mg/g upon increasing the concentration of NaCl from 0.05 to 0.1 M. Above 0.1 M NaCl, the removal % remains constant. This means that the presence of Na⁺ ions helps to neutralize or “shield” the negative charges on the dye’s molecules, thereby reducing electrostatic repulsion. For CV, the removal % increased from from 62.2 to 68.34 mg/g upon increasing the concentration of Na_2_CO_3_ from 0.05 to 0.1 M. Above 0.1 M Na_2_CO_3_, the removal % remains constant. This means that Na_2_CO_3_ creates optimal chemical conditions for improved dye removal through enhanced surface interaction by making the solution more alkaline and developing a more negatively charged surface that enhances electrostatic attraction with the positively charged CV molecules, leading to increased adsorption [67, 68].

#### Desorption and regeneration

**Fig. 5:**
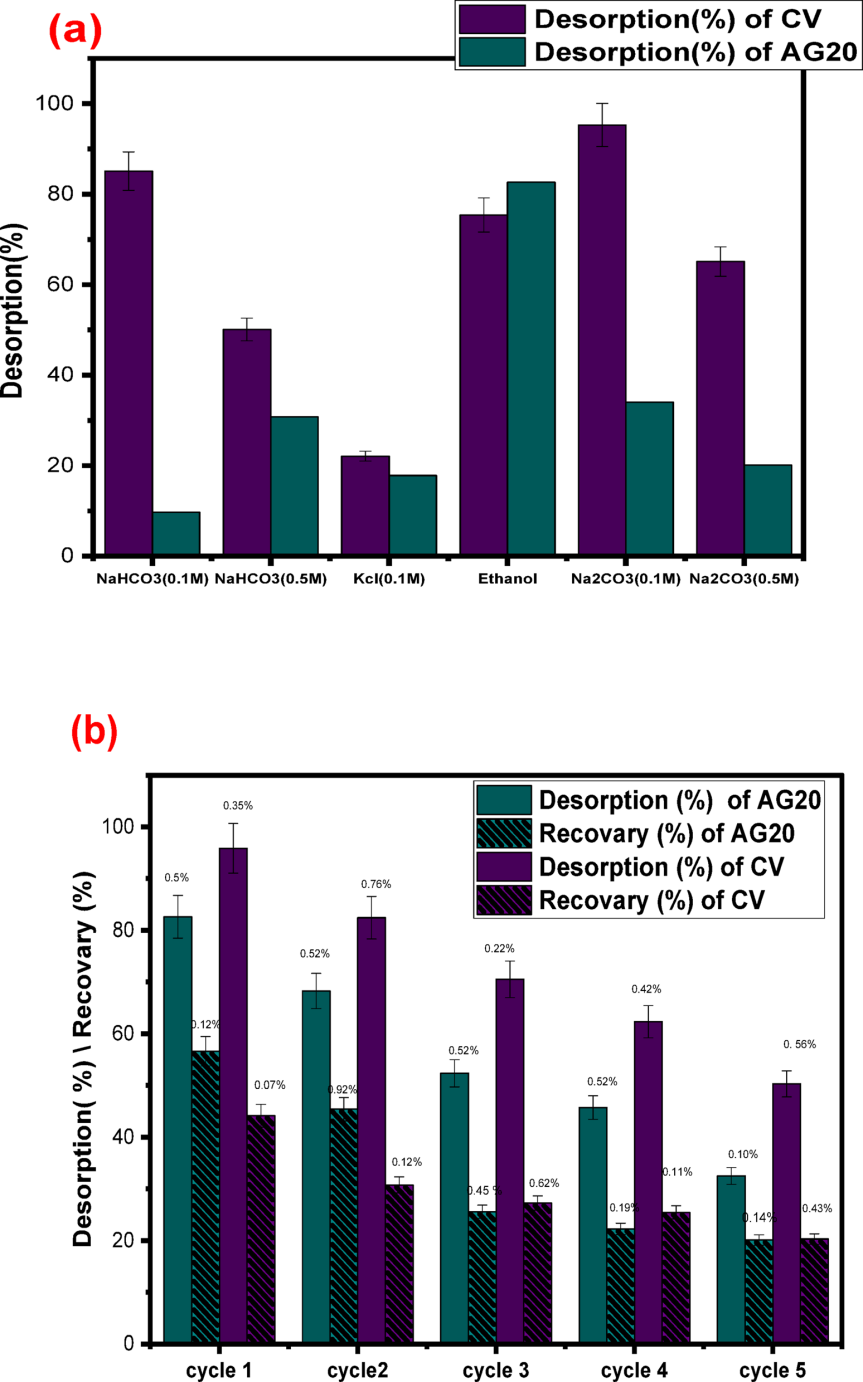
**a** Desorption of CV and AG2O from CK biosorbent by different eluents, **b** Repeated five cycles of AG20 and CV adsorption–desorption using ethanol for AG20 and Na_2_CO_3_ (0.1 M) to CV as eluents, (*n* = 5) *RSD relative standard deviation

To assess the regeneration capacity of the CK biosorbent, desorption experiments were conducted under optimal conditions using various eluents such as NaHCO₃ (0.1 M and 0.5 M), Na₂CO₃ (0.1 M and 0.5 M), ethanol (99%), and KCl (0.1 M), as shown in Fig. 5a. Ethanol (99%) was chosen for desorption of AG20 due to its high affinity for dissolving anionic dye molecules. Na₂CO₃ (0.1 M) was used for desorption CV to weaken electrostatic interactions between the cationic dye and the surface of biosorbent [69, 70].

Under optimized conditions, five consecutive adsorption-desorption cycles were performed for AG20 and CV to assess the long-term reusability and stability of CK biosorbent .

The relative standard deviation (RSD), a statistical measure of precision, was calculated for each cycle to assess the performance consistency of CK biosorbent. As shown in Fig. 5b, the low RSD values obtained across all cycles demonstrate that CK maintains a stable and reproducible adsorption efficiency, confirming its practical potential as a reusable and regenerable biosorbent for dye removal in multiple-cycle operations.

#### pH-responsive removal of AG20 and CV in a mixture

**Fig. 6:**
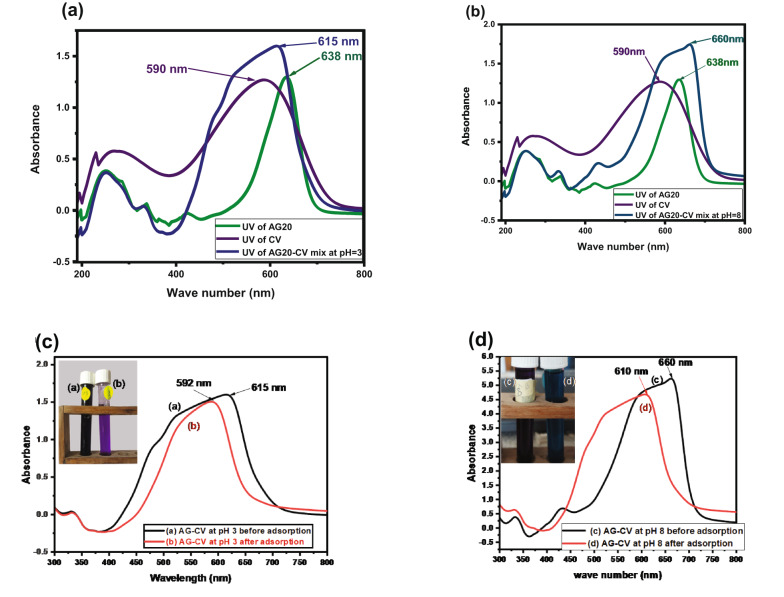
UV data of **a** AG20-CV mixture at pH = 3 is compared with AG20 and CV, **b** CV-AG20 mixture at pH = 8 is compared with CV and AG20, **c** CV-AG20 mixture at pH = 3, **d** CV-AG20 mixture after adsorption by CK

The measured maximum absorbance wavelengths (λ_max_) are 590 nm and 638 nm for AG20 and CV, respectively. Significant spectral overlap was observed in the mixed-dye system; with new peaks appearing at 615 nm at pH 3 and 660 nm at pH 8, as shown in Fig. 6(a–b). These shifts indicate electronic interactions between the dyes and possible aggregation effects influenced by pH. Additional experiments revealed further λ_max_ shifts, with AG20–CV at pH 3 showing a peak at λ_max_ at 592 nm as shown in Fig. 6c, and at 610 nm at pH of 8 as shown in Fig. 6d. Due to this pronounced overlap, the absorbance of the mixture was recorded as a whole in the pH-responsive removal experiments, and the reported concentrations correspond to the total dye concentration rather than individual dyes. This approach was intended to evaluate the overall removal efficiency of CK biosorbent under mixed-dye conditions that closely simulate real wastewater. All adsorption experiments were conducted at the characteristic λ_max_ of each system.

This approach was intended to evaluate the overall removal efficiency of CK under mixed-dye conditions that closely simulate real wastewater. To simulate these conditions, 0.01 g of CK biosorbent was added to a binary dye solution (100 mg/L of each dye) at pH 3 and 8, followed by shaking at 120 rpm. The equilibrium concentrations of the dyes were measured using UV–Vis spectroscopy, and removal efficiency was calculated via Eq. (2).

Notably, distinct color changes were observed before and after adsorption at different pH values. At pH 3, the solution changed from dark purple to light violet as shown in Fig. 6c, indicating effective adsorption of the anionic AG20 dye, likely due to increased protonation of the CK surface that enhances electrostatic attraction. At pH 8, the color shifted from dark purple to greenish violet as shown in Fig. 6d, suggesting decreased removal efficiency due to surface deprotonation and possible dye repulsion or molecular rearrangement.

Moreover, desorption experiments confirmed that pH adjustment could selectively trigger dye release, with CV and AG20 desorbing under specific pH conditions. This reversible and controlled behaviour strongly supports the classification of CK as a pH-responsive and smart biosorbent, capable of adjusting its adsorption and release performance according to environmental pH stimuli. Such behaviour enhances its potential for practical applications in wastewater treatment, especially in systems containing multiple contaminants.

The absorbance vs. wavelength spectra at different adsorption times for both dyes are provided in Fig. S9(a and b). These graphs clearly demonstrate the gradual decrease in the λ_max_ intensity with time from 0 time to 360 min.

#### Dynamic studies

Column experiments were conducted under the optimal pH and temperature conditions previously determined from batch studies to evaluate the removal performance of AG20 and CV dyes using CK. As presented in Table 8, when 0.01 g of CK was used in a column with a 0.7 cm diameter and a low flow rate, 15 mL of both dyes were removed with efficiencies exceeding 40%. CV exhibited a higher adsorption capacity than AG20 under the same conditions. Furthermore, breakthrough curves were generated under various operational parameters, including biosorbent dose, column diameter, and flow rate [71].

The breakthrough curves were constructed by plotting the normalized concentration (C_t_\C_O_) versus time (min), as presented in Fig. S10. These curves provide insight into the adsorption kinetics and saturation behavior under continuous flow conditions.

For AG20, the effluent concentration began to rise at 15 min, C_t_/C_o_ = 0.001, indicating the initial dye leakage from the column. The breakthrough point, defined at C_t_/C_o_ = 0.0487, was closely approached at 150 min. Subsequently, the dye concentration increased gradually, reaching 0.174 at 240 min, 0.439 at 480 min, and 0.790 at 600 min, indicating near exhaustion.

In contrast, CV exhibited a significantly delayed breakthrough, with no detectable concentration in the effluent until 60 min, C_t_/C_o_= 0.003. Even at 150 min, C_t_/C_o_ remained as low as 0.010, showing strong retention by the biosorbent. The concentration continued to rise gradually, reaching C_t_/C_o_ = 0.032 at 240 min and C_t_/C_o_ = 0.120 at 360 min. At 600 min, CV reached 0.710, showing that complete exhaustion had not yet occurred while the column was approaching saturation.

These observations confirm that CK exhibits a stronger adsorption capacity and longer saturation time for the cationic dye CV than the anionic dye AG20. This difference is primarily attributed to electrostatic interactions: the negatively charged surface of CK favors the adsorption of the positively charged CV, while AG20 may experience repulsion.

Thus, the breakthrough profile validates the selectivity of CK toward cationic species and its potential application in real wastewater treatment systems involving mixed dye pollutants.

**Table 8:** Column studies for removing the AG20 and CV dyes

Effect of dose					
Dye	Biosorbent (g)	Flow rate (mL/min)	Diameter of column (cm)	Removal (%)	Adsorption capacity (mg/g)
	0.005	0.36	0.7	30.2	90.6
	0.01			90.21	135.32
AG20	0.02			90.9	68.18
	0.03			91	45.5
CV	0.005	0.36	0.7	35.3	105.9
	0.01			96.52	144.78
	0.02			97.21	72.91
	0.03			97.38	48.69
*Effect of diameter*					
AG20	0.01	0.36	0.3	76.13	114.19
			0.7	90.21	135.32
			0.9	81.6	120.9
CV	0.01	0.36	0.3	78.34	116.01
			0.7	96.52	144.78
			0.9	89.1	133.65
*Flow rate effect*					
AG20	0.01	0.45	0.7	88.98	131.97
		0.36		90.21	135.32
		0.27		83.59	125.39
CV	0.01	0.45	0.7	93.6	140.4
		0.36		96.52	144.78
		0.27		90.38	135.57

#### Application

The efficiency of CK biosorbent for the removal of both cationic and anionic dyes was evaluated using real water samples under the previously optimized experimental conditions. Calibration curves were established using standard dye solutions (1.0 L) that were prepared under these conditions. Three real samples were tested viz. tap water collected from the laboratory at Mansoura University, wastewater and seawater obtained from Damietta City. The results are shown in Table 9. The recoveries obtained were in the range of 91.3 to 99.78%. The results show that the CK biosorbent could effectively remove anionic and cationic dyes from real water samples.

**Table 9:** Recovery of AG20 and CV dyes from water samples using CK biosorbent (*n* = 5)

Water sample	Dye	Spiked (µg/mL^− 1^)	Measured (µg/mL^− 1^)	Recovered (µgmL^− 1^)	Recovery (%)	RSD (%)
Tap water	AG20	0.00	0.00	0.00	0.00%	–
		50	5.89	40.21	93.4%	0.5
		100	19.2	80.6	93.9%	0.78
	CV	0.00	0.00	0.00	0.00%	–
		50	20.1	29.9	97.9%	0.37
		100	39.2	60.8	99.51%	0.98
Sea water	AG20	0.00	0.00	0.00	0.00%	–
		50	7.76	42.24	98.12%	1.14
		100	14.09	85.91	99.78%	0.67
	CV	0.00	0.00	0.00	0.00%	–
		50	22.12	27.88	91.3%	0.27
		100	40.37	59.63	96.6%	1.01
Waste water	AG20	0.00	0.00	0.00	0.00%	-
		50	8.65	41.35	96.05%	0.55
		100	15.5	84.5	98.14%	0.39
	CV	0.00	0.00	0.00	0.00%	–
		50	21.55	28.45	93.13%	1.08
		100	41.3	58.7	96.07%	0.93

#### Performance of the CK biosorbent

For enhancing the value of CK biosorbent, a comparative study of the maximum adsorption capacity of CK biosorbent for AG20 and CV to other adsorbents in the literature was carried out as presented in Table 10. It was found that CK biosorbent is comparable or better than other mentioned adsorbents for the removal of the AG20 and CV dyes. The morphological properties like structure, surface area, and functional groups of each adsorbent are the main reasons for the difference in the AG20 and CV uptake values.

**Table 10:** Comparison of the maximum sorption capacity of CV and AG20 by the CK biosorbent with some of the previously published articles

Adsorbent	q_e_ (mg/g)	Dye	References
Palm kernel shell-derived biochar (BC-PKS)	24.45	CV	[72]
Peanut husk (PH)	20.95	CV	[73]
The avocado pear seed activated carbon (APSAC)	3.3254	CV	[74]
Adsorption and removal of crystal violet dye from aqueous solution by modified rice husk	90.02	CV	[75]
Decontamination of crystal violet using a nanocomposite adsorbent based on pine cone biochar modified with CoFe2O4/Mn-Fe LDH	98.85	CV	[76]
Corn Kernel biosorbent (CK)	61.1	CV	This study
Modified sepiolite, hexadecyltrimethylammonium-modified sepiolite (HDTMA-Sep)	58	AG20	[77]
Modification of Nano-zeolite Clinoptilolite by iron oxide Magnetic (Nano zeolite-Fe_3_O_4_)	49.7	AG20	[78]
Pine tree-derived biochar	16.52	AG20	[79]
Corn Kernel biosorbent (CK)	85.74	AAG20	This study

#### Plausible mechanism of adsorption of AG20 and CV onto CK

**Fig. 7:**
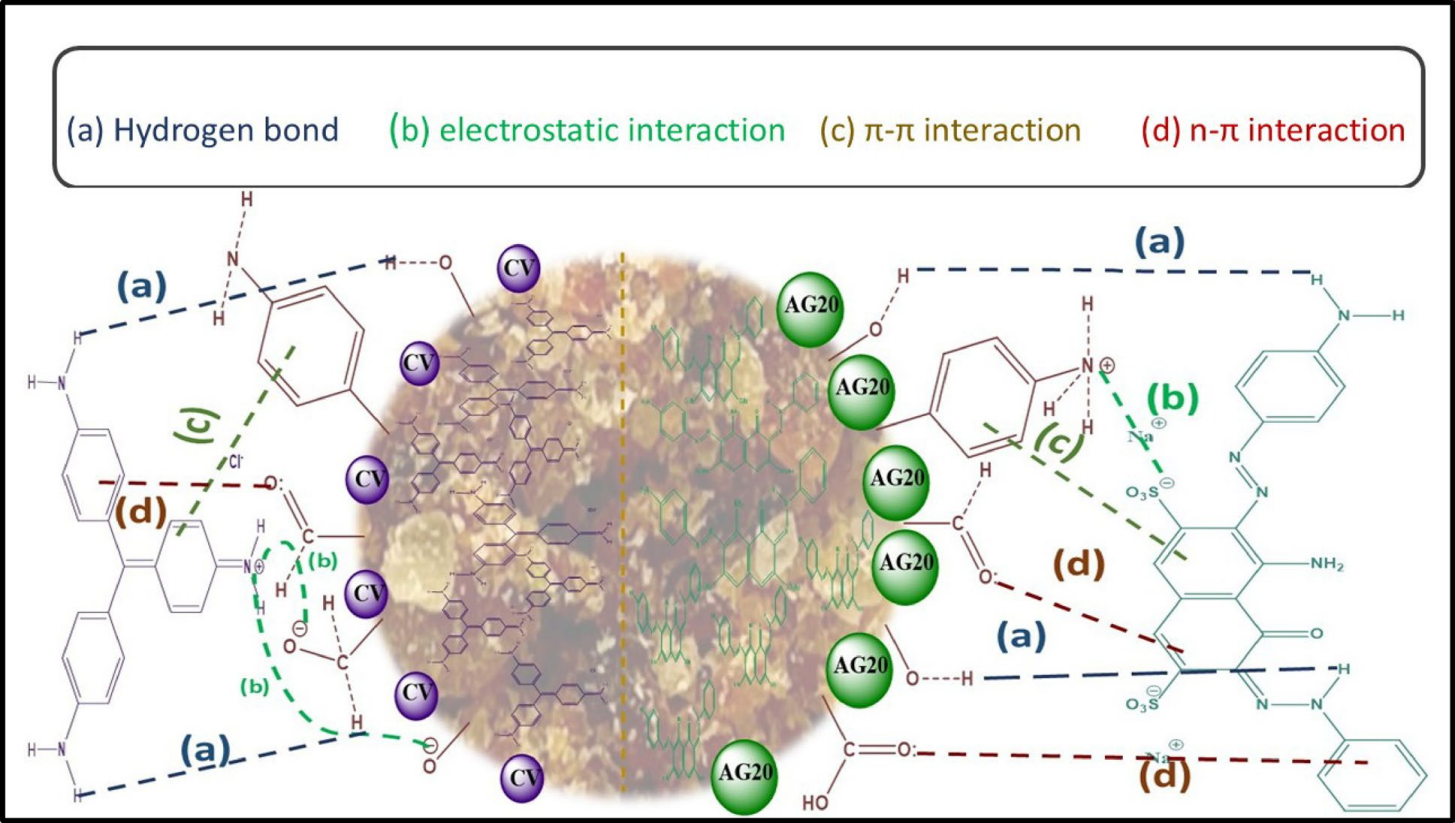
Schematic illustration of AG20 and CV dyes adsorption on the CK biosorbent surface

The FTIR analysis confirms that the CK biosorbent surface is rich in functional groups, including phenyl aniline, carboxyl (–COOH), aldehyde (–CHO), and hydroxyl (–OH), which serve as active sites for dye adsorption. The FTIR spectra obtained after the cationic dye (CV) adsorption and anionic dye (AG20) illustrate the underlying adsorption mechanisms. Additionally, the optical images presented in Fig. 1 and the interaction schematic in Fig. 7 provide visual support for the proposed mechanisms governing the adsorption of AG20 and CV onto CK. These findings confirm that the functional groups on CK actively participated in adsorption.

The adsorbates (AG20 and CV) and the biosorbent interact through several mechanisms, including hydrogen bonding, electrostatic interactions, π–π stacking, n–π interactions, and pore diffusion. These five distinct interaction pathways collectively contribute to the high affinity of CK for both dye molecules. Although multiple interaction types are involved, the low mean adsorption energies (E = 1.085–1.183 kJ/mol from the D–R model) and the absence of covalent bond formation confirm that the adsorption mechanism is dominated by physisorption rather than chemisorption. This agrees with previous reports showing that π–π stacking, hydrogen bonding, and electrostatic forces can occur in physisorption systems on homogeneous surfaces. Importantly, there is no contradiction between the process’s physisorption nature and the Langmuir isotherm’s suitability in describing the adsorption behavior. The Langmuir model reflects monolayer adsorption on homogeneous sites and does not inherently imply chemisorption; it can successfully describe physisorption systems [80].

1. Pore diffusion
  Pore diffusion is critical in controlling the adsorption mechanism, as demonstrated by the second stage of the (IPD) model. The CK biosorbent has an average pore diameter of 46.67 Å, significantly larger than the molecular diameters of AG20 (20–25 Å) and CV (14 Å). This indicates that the pores are sufficiently broad to diffuse both dye molecules effectively. Such a favorable size difference facilitates IPD, allowing dyes to access the internal porous structure with minimal steric hindrance and enhancing the availability of interior adsorption sites. Furthermore, SEM images provide additional support for this mechanism, as post-adsorption surfaces appeared smoother and more compact, with pore visibility implying that dye molecules had filled or blocked the surface pores following successful adsorption. The observed morphological compactness confirms strong interactions between the dyes and the CK surface, which aligns with a physicochemical adsorption process dominated by pore diffusion.

2. Hydrogen bonding
  The interaction occurs between the hydrogen donors (–OH, –COOH, –CHO groups) on the CK surface and the nitrogen atoms within the AG20 and CV dye molecules. This is supported by the broad IR absorption band around 3300–3400 cm⁻¹ and subtle shifts observed after dye loading, confirming the formation of hydrogen bonds. These bonds are weak, reversible, and characteristic of physisorption processes.

3. n–π interactions
  It occurs between lone pair electrons (n) on the oxygen atoms of hydroxyl and carboxyl groups and the π-electron system of the dye’s aromatic structures, particularly evident in the presence of functionalized nitrogens and sulfonated aromatic backbones.

4. Electrostatic interaction
  AG20 adsorption occurred at pH < pH_PZC_, resulting in positively charged CK surface sites (e.g., NH₃⁺), which interact with the negatively charged sulfonate groups (–SO₃⁻) of AG20. Conversely, CV adsorption occurred at pH > pH_PZC_, generating a negatively charged CK surface and enabling interaction with the positively charged quaternary ammonium group of CV. These complementary charges facilitate strong electrostatic.

5. π–π interactions
  occurred between the aromatic rings of AG20 and CV and the phenyl aniline residues in the CK surface (as confirmed by the FTIR band shifts around 1600–1500 cm⁻¹). This stacking enhances adsorption affinity through π-electron cloud interactions.

Although multiple interaction types are involved, the low mean adsorption energies (E = 1.085–1.183 kJ/mol from the D–R model) and the absence of covalent bond formation confirm that the adsorption mechanism is dominated by physisorption rather than chemisorption. This agrees with previous reports showing that π–π stacking, hydrogen bonding, and electrostatic forces can occur in physisorption systems on homogeneous surfaces.

### Toxicity, leaching, and environmental fate studies of CK before and after dye binding (CV-CK and AG20-CK)

It is important to study the environmental safety and compatibility of developed adsorbents; hence, the toxicity of CK before and after binding (CV- and AG20-CK) on two types of bacteria, viz. Gm + ve *(St. aureus)* and Gm-ve bacteria *(E.coli)* were investigated. The results, Table 11, confirmed that the raw and dye-loaded CK had no antibacterial activity against the two tested bacteria in water medium. Therefore, the developed CK adsorbent is considered to have no toxic effect, ensuring its safety when applied to actual wastewater processing.

The prepared MC’s water solubility was also investigated to study CK’s leaching before and after binding (CV- and AG20-CK). There was no noted decrease in the overall mass of CK, CV-CK, or AG20-CK, indicating the water insolubility of the prepared samples. A finding that denotes the difficult leaching of CK post adsorption.

**Table 11:** Toxicity studies of pristine CK and dye-loaded CK post adsorption

Compound	IZD (mm)	
	Gm-ve (E. coli)	Gm + ve (S. aureus)
CK	0.00	0.00
CK-CV	0.00	0.00
CK-AG20	0.00	0.00
Amoxicillin	5	22

The spent CK adsorbents can be easily recycled for the circular economy through chemical Desorption using ethanol (99%) for the Desorption of AG20 and Na₂CO₃ (0.1 M) for the Desorption of CV. The regenerated samples perform remarkably well as adsorbents in wastewater adsorption. The reuse of the spent adsorbents can not only benefit the environment but also reduce the overall cost of the application. Under optimized conditions, long-term reusability and stability were assessed by performing five consecutive adsorption-desorption cycles for AG20 and CV. The results demonstrated that CK maintains a stable and reproducible adsorption efficiency, confirming its practical potential as a reusable and regenerable biosorbent for dye removal in multiple-cycle operations, as discussed in section.

## Conclusion

This study demonstrates that CK is an effective and sustainable biosorbent for removing anionic (AG20) and cationic (CV) dyes. Characterization techniques, including elemental Analysis, TGA, SEM, BET (with an average pore size of 46.67 Å), pHpzc (5.9), and FTIR, confirmed the presence of abundant surface functional groups (–OH, –COOH, –CHO, and phenyl aniline) suitable for dye adsorption. The adsorption performance of AG20 and CV was influenced by solution pH, initial dye concentration, temperature, and contact time. Equilibrium data fitted well with the Langmuir isotherm model, while the kinetics followed a pseudo-second-order model. Thermodynamic parameters (negative ΔG° and positive ΔH°) indicated that the adsorption process is spontaneous and endothermic. Statistical evaluation supported model validity using χ², MSE, SSE, and hybrid error analysis. CK achieved a maximum adsorption capacity of 85.74 mg/g for AG20 and 61.1 mg/g for CV within 120 min. The CK biosorbent was successfully applied to remove AG20 and CV dyes from real water samples in batch and column modes. Moreover, the CK biosorbent demonstrated selective adsorption capability in mixed dye system. Regeneration studies showed that ethanol was the most effective desorbing agent for AG20, while 0.1 M Na₂CO₃ was optimal for CV, with CK maintaining excellent performance over five adsorption–desorption cycles. The proposed adsorption mechanisms involve pore diffusion, electrostatic interactions, hydrogen bonding, and n–π interactions between dye molecules and functional groups on the CK surface. Toxicity tests on two types of bacteria were also performed for CK before and after dye binding. No toxic effects were observed. The eco-friendly adsorbent developed in this study can be used for treatment of wastewater.

## Supplementary Information

Below is the link to the electronic supplementary material.

Supplementary Material 1

## Data Availability

Data is provided within the manuscript or supplementary information files.
