# Supplementary material for: Pristine corn kernels as a pH-responsive biosorbent for selective removal of cationic and anionic dyes
Source: BMC Chem. 2025 Oct 29;19(1):292. doi: 10.1186/s13065-025-01649-1 (PMC12574273; doi:10.1186/s13065-025-01649-1)
Supplement: Supplementary file 1 — Supplementary Material 1 [file 13065_2025_1649_MOESM1_ESM.docx]

**Pristine Corn Kernels as a pH-Responsive Biosorbent for Selective Removal of Cationic and Anionic Dyes**

**Noha A Abd-Rabo^1^, Asmaa A Serage^1^, Elsayed RH El-Gharkawy^1^and Magda A Akl^1^***

^1^Department of Chemistry, Faculty of Science, Mansoura University, Mansoura 35516, Egypt

***Corresponding author**

**Prof Magda A Akl**

**e.mail: [magdaakl@yahoo.com](mailto:magdaakl@yahoo.com)**

| 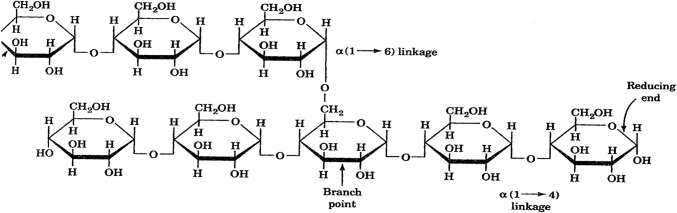  (a)Structure of starch |
| --- |
| 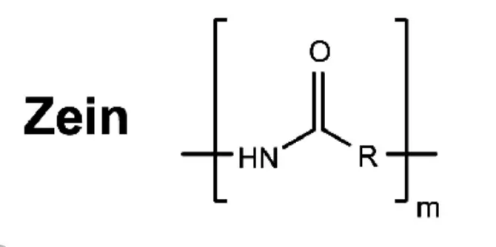  (b) Zein maize protein |

**Figure S1:** Structure of **(a)** starch and **(b)** zein maize protein of corn kernel.

**Figure S2**: The removal percentages of dyes by different parts of corn .

**Figure S3:** Linear BET plots of nitrogen adsorption isotherms at 77.35^o^K

for CK biosorbent.

**Figure S4**: The pH_PZC_ value of CK**.**

|  |  |
| --- | --- |
|  |  |
|  | |

**Figure S5:**  **(a)** linear Langmuir for AG20, and CV adsorption, **(b)** nonlinear Langmuir for AG20, and CV adsorption, **(c)** linear Freundlich for AG20, and CV adsorption, **(d)** nonlinear Freundlich for AG20, and CV  adsorption, and **(e)** D-R for AG20 and CV adsorption.

|  |  |
| --- | --- |
|  |  |
|  | |

**Figure S6 :(a)** linear PFO for AG20, and CV adsorption, **(b)** nonlinear PFO for AG20, and CV adsorption, **(c)** linear PSO for AG20, and CV adsorption, **( d)** nonlinear PSO for AG20, and CV  adsorption, and **(e)** IPD for AG20 and CV adsorption.

| **** | **** |
| --- | --- |

**Figure S7:** Plot of ln K_C_ versus (1/T) absolute temperature for the adsorption of (lnK_c_vs 1/T) for the adsorption of **(a)** AG20, and **(b)**CV dye onto CK.

| **** |
| --- |
| **** |

**Figure S8: (a)** Effect of ionic strength on AG20 and CV, and **(b)** the influence of NaCl CK-AG20 and Na_2_CO_3_ on CK-CV at different concentrations.

|  |  |
| --- | --- |

**Figure S9:** Absorbance vs Wavelength spectra at different time intervals for **(a)** AG 20 and **(b)** CV.

**Figure S10:** breakthrough curve for AG20, and CV.

**Table S1:** FTIR characteristic peaks .

| **Peak range (cm⁻¹)** | **Function groups** | **Interpretation** |
| --- | --- | --- |
| \| **~3400 cm⁻¹-3200 cm⁻¹** \|  \|  \| \| --- \| --- \| --- \| | O–H stretching (broad band) | Hydrogen bonding in starch, carboxyl groups |
| \| **~2925 cm⁻¹-2859 cm⁻¹** \|  \|  \| \| --- \| --- \| --- \| | C–H stretching (asymmetric CH₂, CH₃) | Aliphatic chains (starch/zein maize proteins) |
| \| **~1741 cm⁻¹** \|  \|  \| \| --- \| --- \| --- \| | C=O stretching (carbonyl) | \|  \| \| --- \|  \| Esters/lipids/polysaccharides \| \| --- \| |
| \| **~1636 cm⁻¹** \|  \|  \| \| --- \| --- \| --- \| | Amide I (C=O stretch of proteins) | Zein maize protein |
| \| **~1545 cm⁻¹** \|  \|  \| \| --- \| --- \| --- \| | Amide II (N–H bending + C–N stretching) | Zein maize protein |
| \| **~1491 cm⁻¹** \|  \|  \| \| --- \| --- \| --- \| | Aromatic C=C | Phenylalanine |
| **~1400** **cm⁻¹**–**1300** **cm⁻¹** | Various peptide bands | Protein conformation (Amide III) |
| **~1346** **cm⁻¹** -**1314** **cm⁻¹** | \|  \| Symmetric/asymmetric S=O stretching (SO₃⁴⁻ sulfate group) \| \| --- \| --- \| | Interaction with AG20 dye |
| \| **~1050 cm⁻¹–1030 cm⁻¹** \|  \|  \| \| --- \| --- \| --- \| | C–O–C stretching (ether) | Glycosidic linkages in starch |
| **~** **926 cm⁻**¹ | C–H bending (polysaccharide skeletal band) | Corn starch |
| **~1635** **cm⁻¹**-**1587** **cm⁻¹** | Shifted Amide I / II / C=O / N–H peaks | Evidence of CV interaction with CK |
| **~1664** **cm⁻¹**- **1611** **cm⁻¹** | Shifted Amide I / II / C=O / N–H peaks | Evidence of AG20 interaction with CK |
| **~** **914 cm⁻**¹ | Shifted C–H bending (polysaccharide skeletal band) | Evidence of CV interaction with CK |
| **~** **below 900 cm⁻**¹ | Shifted C–H bending (polysaccharide skeletal band) | Evidence of AG20 interaction with CK |
